# Regional Changes in the Sequence of Cotton Leaf Curl Multan Betasatellite

**DOI:** 10.3390/v6052186

**Published:** 2014-05-23

**Authors:** Sohail Akhtar, Muhammad Nouman Tahir, Ghulam Rasool Baloch, Shaista Javaid, Ali Qaiser Khan, Imran Amin, Rob W. Briddon, Shahid Mansoor

**Affiliations:** Agricultural Biotechnology Division, National Institute for Biotechnology and Genetic Engineering, Faisalabad, Pakistan; E-Mails: sakhtar.pp@uaf.edu.pk (S.A.); nouman.nibge@yahoo.com (M.N.T.); grasoolbaloch@yahoo.com (G.R.B.); shaistajavaid85@gmail.com (S.J.); kk_684@yahoo.com (A.Q.K.); imranamin1@yahoo.com (I.A.); rob.briddon@gmail.com (R.W.B.)

**Keywords:** begomovirus, betasatellite, genetic diversity, recombination

## Abstract

Cotton leaf curl disease (CLCuD) in Pakistan and northwestern India is caused by monopartite begomoviruses in association with an essential, disease-specific satellite, Cotton leaf curl Multan betasatellite (CLCuMB). Following a recent upsurge in CLCuD problems in Sindh province (southern Pakistan), sequences of clones of CLCuMB were obtained from Sindh and Punjab province (central Pakistan), where CLCuD has been a problem since the mid-1980s. The sequences were compared to all sequences of CLCuMB available in the databases. Analysis of the sequences shows extensive sequence variation in CLCuMB, most likely resulting from recombination. The range of sequence variants differ between Sindh, the Punjab and northwestern India. The possible significance of the findings with respect to movement of the CLCuD between the three regions is discussed. Additionally, the lack of sequence variation within the only coding sequence of CLCuMB suggests that the betasatellite is not involved in resistance breaking which became a problem after 2001 in the Punjab and subsequently also in northwestern India.

## 1. Introduction

Cotton leaf curl disease (CLCuD) is a major constraint for cotton production across central Pakistan and northwestern areas of India [1]. The disease appeared in an epidemic form in the early 1990s in the vicinity of the city of Multan, Pakistan [2]. In the late 1990’s the introduction of resistant cotton varieties developed by conventional breeding methods restored Pakistani cotton production to pre-epidemic levels. Unfortunately, from 2001 onwards, the disease reappeared on all previously resistant varieties in the vicinity of the city of Burewala and rapidly spread across most cotton growing areas of Pakistan and into northwestern India [3,4]. CLCuD is caused by begomoviruses in association with a disease-specific satellite, Cotton leaf curl Multan betasatellite (CLCuMB) [5,6].

Viruses of the genus *Begomovirus* (family *Geminiviridae*) are transmitted by the whitefly *Bemisia tabaci*, with genomes consisting of either one or two small (~2.8 kb), circular single-stranded (ss)DNA components encapsidated in twinned, quasi-isometric particles [7]. In the Old World (OW) a small number of bipartite begomoviruses have been identified, the majority instead being monopartite. This contrasts with the New World where begomoviruses with bipartite genomes are the norm; the first native monopartite begomovirus only having been identified recently [8,9]. In the OW the majority of monopartite are associated with a class of ssDNA satellites collectively known as betasatellites [10,11].

Betasatellites are small (~1.4 kb), circular, ssDNA satellites that have only been identified in the OW, are associated with monopartite begomoviruses, and depend upon a helper virus for replication, movement in and transmission between plants [6,12,13]. The sequences of betasatellites have three major features—a single conserved (in both position and sequence) gene (referred to as βC1), a region of sequence rich in adenine (A-rich) and a sequence conserved among all betasatellites (known as the satellite conserved region (SCR) [12,14]. The SCR contains a predicted stem-loop structure containing the nonanucleotide sequence TAATATTAC which, for geminiviruses, is the origin of virion-strand DNA replication [15]. The βC1 gene typically encodes a protein of 118 amino acids that mediates all betasatellite functions. βC1 is a pathogenicity determinant, a suppressor of post-transcriptional gene silencing (PTGS; a small RNA-mediated host defense), binds DNA and possibly mediates virus movement in plants [16,17,18,19,20,21]. The function of the A-rich region is unclear. It has been suggested that this may be a “stuffer” to increase the size of the molecule for encapsidation [12,22].

CLCuD across central Pakistan and northwestern India during the 1990s was associated with at least six distinct monopartite begomoviruses but only a single betasatellite, referred to as the “Multan” strain of CLCuMB (CLCuMB^Mul^) [6]. Following resistance breaking, the disease across central Pakistan was found to be associated with only a single virus, a recombinant species, *Cotton leaf curl Burewala virus* (CLCuBuV), consisting of sequences derived from two virus species present in cotton before resistance breaking [23]. The betasatellite associated with CLCuBuV was also recombinant, with a small replacement of sequences within the SCR derived from a distinct betasatellite, Tomato leaf curl betasatellite (ToLCB) [23,24]. This is now referred to as the “Burewala” strain of CLCuMB (CLCuMB^Bur^).

Until recently CLCuD was only a minor, sporadic problem across central and southern Sindh province, Pakistan. For this reason CLCuD resistant cotton varieties were not widely grown—farmers having to make a choice between the high yielding, but susceptible varieties, or playing safe with the lower yielding resistant varieties. In 2005 there was an upsurge of CLCuD in Sindh which was shown to be associated with a virus species present in cotton in the Punjab pre-resistance breaking, a new recombinant virus distinct from CLCuBuV, CLCuMB^Bur^ and a new strain of CLCuMB containing a smaller recombinant fragment from ToLCB, now referred to as the Shahdadpur strain of CLCuMB (CLCuMB^Sha^) [25]. In northwestern India two recent studies have shown CLCuBuV to dominate in cotton post-resistance breaking but a virus common prior to resistance breaking, *Cotton leaf curl Rajasthan virus* (CLCuRaV) was also detected infrequently [4,26]. Although both studies showed the presence of CLCuMB, neither determined the strain thereof.

The study presented here has analyzed the sequences of CLCuMB recently isolated from Sindh and the Punjab (Pakistan), for comparison to isolates obtained earlier, and has identified region-specific sequence changes. The significance of these findings is discussed.

## 2. Materials and Methods

### 2.1. Sample Collection

Leaves of cotton plants showing symptoms typical of CLCuD were collected from fields around the Central Cotton Research Institute (CCRI) Multan (Punjab, Pakistan) in 2008/2009 and areas of Sindh in 2010 sampled in previous studies [25,27].

### 2.2. PCR-Mediated Amplification and Cloning of Betasatellites

Total genomic DNA was extracted from leaves using the CTAB method [28] and stored at −20 °C. Betasatellites were amplified by PCR using universal primers [29]. PCR products were cloned in pTZ57R/T (Fermentas) and sequenced commercially, in both orientations, using the primer walking strategy (Macrogen Inc., Seoul, Korea).

### 2.3. Sequence Analysis

Sequences were assembled and analysed using the Lasergene (DNASTAR Inc., Madison, Wisconsin, USA) sequence analysis package. Sequence similarity searches (BLAST) were performed online by comparing the sequences characterized in this study with sequences available in the databases using BLAST. Multiple sequence alignments were performed using MegAlign (Lasergene) and ClustalX2 [30]. Phylogenetic dendrograms were constructed using the Neighbour-joining algorithm of ClustalX2 and viewed, manipulated and printed using Treeview [31]. Potentially recombinant sequences were identified using the Recombination Detection Program version 3 (RDP version 3; [32]).

## 3. Results

### 3.1. Analysis of Betasatellite Sequences

A total of 11 presumed full-length (~1.4 kb; 4 from the Punjab and 7 from Sindh) and 26 defective (~0.7 kb; 2 from the Punjab and 24 from Sindh) betasatellites were cloned and sequenced. The sequences are available in the nucleotide sequence databases under the accession numbers given in Table 1 and Table 2. The complete nucleotide sequences of betasatellites were compared to sequences of betasatellites available in the database using BLAST. The comparison revealed sequence identities ranging from 96% to 99% with available isolates of CLCuMB. Since the species demarcation threshold for betasatellite is 78% [33], this indicates that all betasatellites cloned from cotton here are isolates of CLCuMB.

**Table 1:** Origins and features of full-length betasatellites.

Isolate descriptor	Origin	Accession number	Size (nt)	Position of βC1 gene (coordinates of start/stop codons)
[PK:Hala:09]	Hala/Sindh	HE601944	1350	195–551
[PK:Hala1:09]	Hala/Sindh	HE601945	1350	195–551
[PK:Sha:06]	Shahdadpur/Sindh	HE601941	1375	195–551
[PK:Tjam2:09]	Tandojam/Sindh	HE601946	1364	195–551
[PK:Tjam1:09]	Tandojam/Sindh	HE601947	1348	195–551
[PK:Tjam:09]	Tandojam/Sindh	HE601948	1349	194–550
[PK:Tjam:06]	Tandojam/Sindh	HE601940	1350	195–551
[PK:Mul:08]	Multan/Punjab	HE601938	1345	195–551
[PK:Mul1:08]	Multan/Punjab	HE601939	1349	195–551
[PK:Mul1:09]	Multan/Punjab	HE601942	1350	195–551
[PK:Mul2:09]	Multan/Punjab	HE601943	1355	195–551

**Figure 1:**
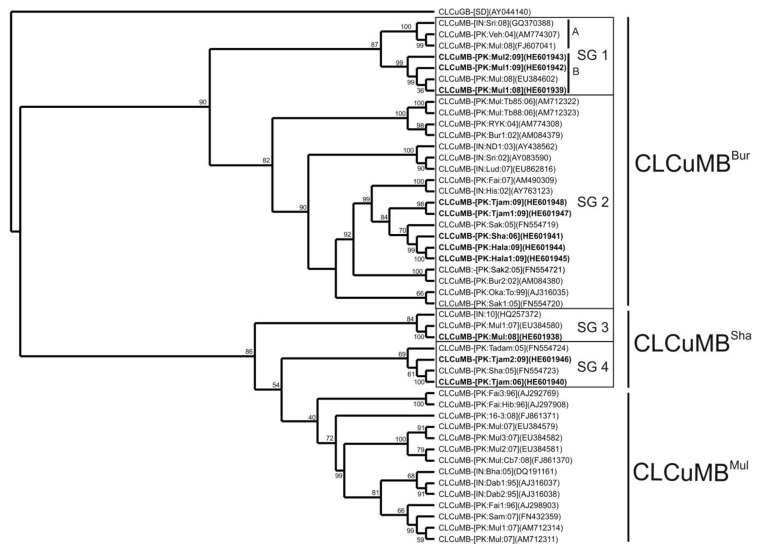
Phylogenetic analyses of Cotton leaf curl Multan betasatellite (CLCuMB) sequences. Shown is a neighbor-joining phylogenetic dendrogram based upon alignment of the complete nucleotide sequences of CLCuMB isolates produced as part of the study presented here (highlighted with bold text) with selected CLCuMB sequences available in the databases. The alignment was arbitrarily rooted on the sequence of Cotton leaf curl Gezira betasatellite (CLCuGB-[SD]), a distantly related betasatellite originating from Sudan, as an out group. In each case, the isolate descriptor and accession number is given. Numbers at the nodes are percentage bootstrap confidence values (1000 replicates). The recombinant CLCuMB^Bur^, the recombinant CLCuMB^Sha^ and non-recombinant CLCuMB^Mul^ isolates are indicated. The subgroups (SG) are discussed in the text.

A phylogenetic analysis, based upon alignment of the full-length sequences determined here with available CLCuMB sequences in the databases in shown in Figure 1. The dendrogram shows the satellites to segregate into two major groups. The first group contains the non-recombinant CLCuMB associated with the CLCuD epidemic of the 1990s (CLCuMB^Mul^) first identified by Briddon *et al.* [5], as well as the recombinant CLCuMB recently identified in the Sindh province (Pakistan; CLCuMB^Sha^; [25]). Parts of group 1 are also some CLCuMB isolates, labeled subgroup 3 (SG3) in Figure 1, which group with the CLCuMB^Mul^/CLCuMB^Sha^ isolates but are basal to them. The second group contains the CLCuMB sequences first identified with resistance breaking in cotton identified by Amin *et al.* [24], known as the Burewala strain (CLCuMB^Bur^). The CLCuMB isolates characterized as part of the study here fall into both major groups, although none segregate with the non-recombinant CLCuMB^Mul^.

### 3.2. Variation in SCR Sequences

**Figure 2:**
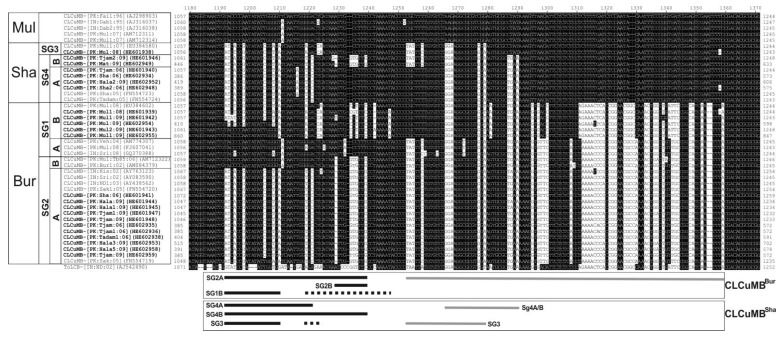
Alignment of the nucleotide sequences of the satellite conserved region (SCR) of CLCuMB isolates derived from the study presented here (highlighted with bold text) with selected sequences from the database. The defective isolates were included in this analysis. The homologous sequence of a Tomato leaf curl betasatellite (ToLCB) isolate was included for comparison. Gaps (-) were introduced into sequences to optimize the alignment. In each case, the isolate descriptor and accession number is given. Sequences differing from CLCuMB-[PK:Fai1:96] (AJ298903; a CLCuMB^Mul^ isolate) are highlighted as black text on a white background. The strain of CLCuMB (Burewala [Bur], Multan [Mul] or Shahdadpur [Sha]) is shown on the left as well as the subgroups (SG) identified in Figure 1. The origins of sequences are shown in the two boxes below the alignment for the recombinant CLCuMB isolates (CLCuMB^Bur^ and CLCuMB^Sha^). Sequences likely originating from ToLCB are shown with grey bars. Sequences homologous to those of CLCuMB^Bur^ are indicated by solid black bars. Sequences of unknown origin are shown by dashed black lines. All other sequences are homologous to CLCuMB^Mul^.

An alignment of part of the SCR sequences of the betasatellite isolates obtained here (including the defective clones) with selected sequences from the databases is shown in Figure 2. The alignment shows the recombinant fragment (~105 nt; grey bar below the diagram), believed to have originated from ToLCB that distinguishes CLCuMB^Bur^ isolates from CLCuMB^Mul^ isolates [23,24]. An RDP analysis (Supplementary Figure S1, Supplementary Table S1) actually suggests that, rather than just spanning the region indicated in Figure 2, the recombination encompasses also the conserved nonanucleotide-containing hairpin for SG1 and SG2A isolates, whereas for SG2B isolates the recombinant fragment is ~150 nt spanning the area indicated in Figure 2. For some of the isolates identified by Amrao *et al.* [25], and also for some isolates identified here, all originating from Sindh, the recombinant fragment of ToLCB is somewhat smaller (~25 nt; grey bar labeled SG4) and was previously designated the “Shahdadpur” strain of CLCuMB (CLCuMB^Sha^). The alignment shows an additional group of CLCuMB isolates that contain a ~27 nt fragment derived from ToLCB that overlaps with the fragment in the SG4A isolates (labeled as SG4B and SG3 in Figure 2). These isolates differ in their origins, the SG4B originating from Sindh and SG3 isolates from the Punjab. The presence of these smaller recombinant fragments of ToLCB is supported by the RDP analysis but only by one of the detection methods (GENECONV; Supplementary Table S1).

**Table 2:** Origins and features of defective betasatellites.

Isolate descriptor	Origin	Accession number	Size (nt)	Coordinates of deleted region *
[PK:Sak:06]	Sakrand/Sindh	HE602942	645	048–758
[PK:Sak1:06]	Sakrand/Sindh	HE602943	690	065–731
[PK:Sak2:06]	Sakrand/Sindh	HE602944	668	065–735
[PK:Sha1:06]	Shahdadpur/Sindh	HE602945	727	096–721
[PK:Hala:09]	Hala/Sindh	HE602950	788	202–763
[PK:Hala1:09]	Hala/Sindh	HE602951	788	202–763
[PK:Hala3:09]	Hala/Sindh	HE602953	818	208–740
[PK:Hala4:09]	Hala/Sindh	HE602956	699	110–763
[PK:Mat1:09]	Matiari/Sindh	HE602957	681	099–786
[PK:Hala5:09]	Hala/Sindh	HE602958	694	097–753
[PK:Tjam:09]	Tandojam/Sindh	HE602959	688	116–780
[PK:Sha:06]	Shahdadpur/Sindh	HE602934	680	157–832
[PK:Sha2:06]	Shahdadpur/Sindh	HE602948	678	130–801
[PK:Hala2:09]	Hala/Sindh	HE602952	712	053–696
[PK:Tjam:06]	Tandojam/Sindh	HE602935	678	129–805
[PK:Tjam1:06]	Tandojam/Sindh	HE602936	678	129–805
[PK:Tadam:06]	Tandoadam/Sindh	HE602937	713	129–769
[PK:Tadam1:06]	Tandoadam/Sindh	HE602938	697	115–771
[PK:TAyar:06]	TandoAllahyar/Sindh	HE602939	680	116–780
[PK:TAyar1:06]	TandoAllahyar/Sindh	HE602940	735	153–773
[PK:TAyar2:06]	TandoAllahyar/Sindh	HE602941	690	084–748
[PK:TAyar3:06]	TandoAllahyar/Sindh	HE602946	685	117–805
[PK:TAyar4:06]	TandoAllahyar/Sindh	HE602947	809	151–714
[PK:Mat:09]	Matiari/Sindh	HE602949	749	115–732
[PK:Mul:09]	Multan/Punjab	HE602954	703	105–754
[PK:Mul1:09]	Multan/Punjab	HE602955	953	224–631

* Relative to isolate CLCuMB-[PK:Sak:05] (FN554719).

The defective (deletion mutant) CLCuMB clones are all approx. half the size (630 to 700 bp) of full-length betasatellites (typically ~1350 bp; [11,12]). The sequences deleted cover the βC1 gene and its promoter. In each the SCR, the A-rich and the sequences between these (across the conserved hairpin structure) are maintained (Table 2).

Not considered in the previous analyses of the SCR sequences of CLCuMB was the sequence immediately upstream of the ToLCB insertions. For the recently characterized isolates, including those identified here, these sequences differ from those of CLCuMB^Mul^. For many of the CLCuMB^Bur^ isolates from Pakistan in the earlier studies [23,24] these sequences are homologous to the those of CLCuMB^Mul^ (the SG1 isolates) characterized earlier. The sequence replacements in this region not homologous to CLCuMB^Mul^ are of two types (shown as either solid black lines or dashed black lines in Figure 2). The origins of the sequences indicated by dashed lines are unclear, having some levels of sequence identity to a number of distinct betasatellite species and could result from recombination, although the RDP analysis did not support this possibility. Overall the isolates segregate, with respect to these replacements, according to the phylogenetic groupings identified in Figure 1. Interestingly the SG2A isolates are exclusively from either Sindh or India whereas the SG2B isolates, with a smaller sequence insertion, come only from the Punjab.

**Figure 3:**
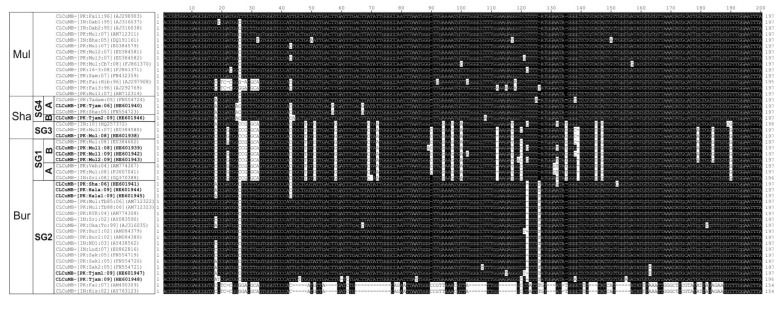
Alignment of the nucleotide sequences immediately downstream of the hairpin structure of CLCuMB isolates derived from the study presented here (highlighted with bold text) with selected sequences from the database. The sequence falls, at least in part, within the SCR. Gaps (-) were introduced into sequences to optimize the alignment. In each case, the isolate descriptor and accession number is given. Sequences differing from CLCuMB-[PK:Fai1:96] (AJ298903; a CLCuMB^Mul^ isolate) are highlighted as black text on a white background. The strain of CLCuMB (Burewala [Bur], Multan [Mul] or Shahdadpur [Sha]) is shown on the left as well as the subgroups (SG) identified in Figure 1.

An alignment of sequences downstream of the hairpin, falls (at least in part) within the SCR [12] of the betasatellite isolates obtained here (including the defective clones) with selected sequences from the databases shown in Figure 3. The alignment shows the CLCuMB^Bur^ SG1 and CLCuMB^Sha^ SG3 isolates share a sequence of *ca*. 123 nt which is distinct from the other isolates. The 123 nt sequence shows no significant levels of sequence identity to other sequences in the databases, is not highlighted by the RDP analysis and its origin thus remains unclear. The isolates with this sequence originate from both regions of Pakistan and northwestern India.

Overall, of 147 recombinant CLCuMB isolates available in the databases, the dominant form in India is SG2A (8 SG1A, 3 SG1B, 32 SG2A and 2 SG4A) whereas SG2B and SG3 dominate in the Punjab (4 SG1A, 15 SG1B, 5 SG2A, 17 SG2B and 18 SG3) and Sindh has only SG2A (31) and SG4 (9 SG4A and 3 SG4B). Sindh, despite the low numbers of CLCuMB sequences available, has variants in common with the Punjab (SG2A), but not the variant common in the Punjab (SG2B), and variants in common with India (SG2A and SG4; SG4 has not been identified in the Punjab).

### 3.3. Analysis of CLCuMB Sequences between the A-Rich Region and the SCR

**Figure 4:**
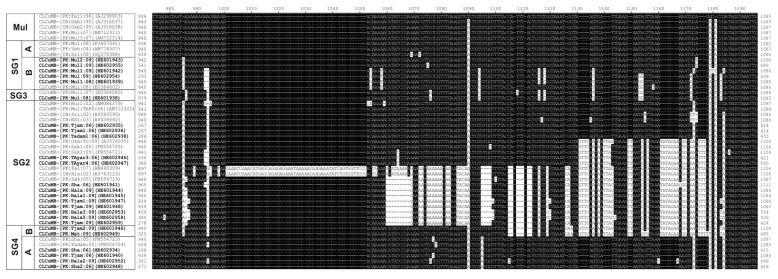
Alignment of the nucleotide sequences of the region between the A-rich and the SCR of CLCuMB isolates derived from the study presented here (highlighted with bold text) with selected sequences from the databases. Gaps (-) were introduced into sequences to optimize the alignment. In each case, the isolate descriptor and accession number is given. Sequences differing from CLCuMB-[PK:Fai1:96] (AJ298903; a CLCuMB^Mul^ isolate) are highlighted as black text on a white background. The sequence groups indicated on the left are those identified in Figure 1.

An alignment of CLCuMB (including the defective clones) sequences between the SCR and A-rich region is shown in Figure 4. This shows two clones (CLCuMB-[PK:Fai:07] AM490309 and CLCuMB-[IN:His:02] AY763123) to have a large insertion/deletion (indel) with respect to the other isolates (coordinates 1000 to 1177 of the alignment in Figure 4). The origin of the insertion is unclear since there is no significant sequence identity to sequences in the database except of the CLCuMB isolates shown here. With the exception of these two clones only CLCuMB isolates originating from Sindh have the indel, although the lengths thereof differ, with the sequence being progressively truncated. Although CLCuMB-[IN:His:02]AY763123 was isolated from cotton in India, CLCuMB-[PK:Fai:07] AM490309 was isolated from tomato and shown to be associated with *Cotton leaf curl Rajasthan virus* [34], a begomovirus shown to be extensively associated with CLCuD in India [4] but not identified in *G. hirsutum* in Pakistan.

### 3.4. Sequence Variation in the Predicted Amino Acid Sequences of βC1

**Figure 5:**
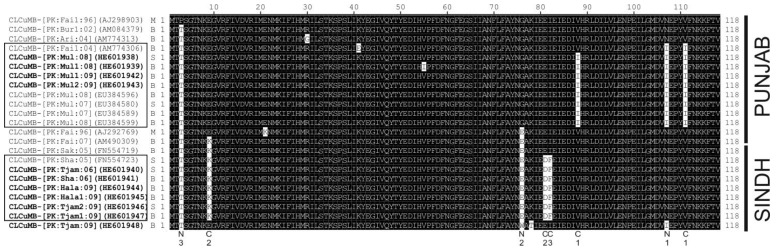
Alignment of the predicted amino acid sequences of the βC1 proteins of selected CLCuMBs. Sequences with the same, or similar, sequence changes have been grouped. In each case, the isolate descriptor, accession number and type (CLCuMB^Bur^ [B], CLCuMB^Mul^ [M], or CLCuMB^Sha^ [S]) are given. Betasatellites produced as part of the study presented here are marked in bold. Sequences are indicated as originating from either the Punjab or Sindh on the right. Standard IUPAC/IUBMB single letter amino acid codes are used. Along the bottom the major amino acid sequence changes are indicated as either conservative (C) or non-conservative (N) with respect to amino acid properties (C1-I and V non-polar/hydrophobic; C2-D,K and E polar/hydrophilic; F and I-non-polar/hydrophobic; N1-I non-polar/hydrophobic whereas N is polar/hydrophilic; N2-G and W non-polar/hydrophobic whereas E is polar/hydrophilic; N3-T polar/hydrophilic whereas P is non-polar/hydrophobic).

An alignment of the predicted amino acid sequences of the βC1 protein of selected CLCuMB isolates is shown in Figure 5, which shows there is little sequence variation in the protein. Most of the major conserved (across multiple isolates and thus likely to be meaningful rather than random sequence variation or sequencing errors) sequence differences are conservative with respect to amino acid properties. Nevertheless, for the majority of isolates there are amino acid changes that differ between isolates originating from the Punjab and Sindh.

## 4. Discussion

CLCuD continues to adversely affect cotton production across Pakistan and northwestern India some 25 years after the first epidemic of the disease. Although resistance to the disease was introduced in the late 1990s, this succumbed quite quickly with the appearance of a resistance breaking strain of the virus complex. The ongoing breeding efforts have identified some sources of tolerance to the resistance breaking strain, but the desired immunity remains elusive [1]. Although the genetic changes in the complex pre- and post-resistance breaking have been determined, the precise mechanism of (molecular basis for) resistance breaking remains unresolved [35]. The betasatellite associated with CLCuD in south Asia, CLCuMB, has been shown to encode the major symptom determinant of the complex [19] and showed significant differences post-resistance breaking, specifically recombination within the SCR [24], which has been used as a genetic marker of the resistance-breaking virus complex.

Cotton is grown widely across the warmer parts of the world. CLCuD occurs in Asia and Africa with distinct etiologies, although in both areas begomovirus-betasatellite complexes are involved [1]. In all areas where cotton is grown the requirements for establishment of CLCuD are present—the main requirement being the presence of the vector *B. tabaci*. There is thus the fear that CLCuD could spread. In at least one instance this fear has been realized, with CLCuD reported for the first time in southeastern China [36]. The study here has assessed differences between the CLCuMB prevalent in two regions of Pakistan (Punjab and Sindh), following a recent upsurge of CLCuD problems in Sindh, and relates this to sequences changes in CLCuMB in the wider region.

For the CLCuD affected plants assessed here, CLCuMB was identified in all instances. This is consistent with the earlier demonstration of the importance of this specific betasatellite for causing the disease in cotton in south Asia [5,37]. The virus, however, appears less important. Experimentally even viruses not usually identified in cotton or associated with CLCuMB can induce CLCuD symptoms in cotton in the presence of CLCuMB—albeit transiently [38]. However, in the absence of CLCuMB even the viruses that do commonly infect cotton show poor infectivity to and atypical symptoms in cotton [5].

For CLCuMB, the sequences between the SCR and the A-rich region have previously been shown to be highly variable [24]. Deletion studies with *Ageratum* yellow vein betasatellite showed that the sequences between the SCR and the A-rich region are important for the *trans*-replication of the betasatellite by the helper begomovirus [39]. This finding was consistent with an earlier study of the *trans*-replication of an unusual betasatellite deletion mutant [40,41]. Nawaz-ul-Rehman *et al*. [41] showed that when *trans*-replicated *in planta* by *Cabbage leaf curl virus*, a virus that is not associated with a betasatellite, mutations occur in the sequences between the SCR and the A-rich region, and levels of betasatellite DNA are higher, suggesting that the sequence changes improve trans-replication. These studies thus indicate that betasatellite sequences between the SCR and the A-rich region are likely constrained by, and change due to, interaction with the helper virus. The changes in these sequences of CLCuMB detected here, with the range of variants in Sindh showing commonalities and differences with both the Punjab and India, are consistent with a greater diversity of helper begomoviruses detected in cotton in Sindh than in the Punjab [23,25,27].

Analysis of the predicted amino acid sequences of the βC1 gene of CLCuMB show that sequence variation in this protein is highly constrained. βC1 is the only gene encoded by betasatellites and mediates all betasatellite functions including suppression of gene silencing, virus movement in plants and modulating host gene expression. These functions are mediated by interaction with host factors, a number of which have been identified [42,43]. This thus indicates that variation in βC1 is constrained by the need to maintain interactions with the host rather than the helper virus.

Analysis of the amino acid sequences of βC1 also seem to indicate that there is no correlation between resistance breaking and βC1 sequence. For example, CLCuMB-[PK:Bur1:02] (AM084379), a “Burewala strain” isolate, lacks the amino acid sequence changes present in the majority of CLCuMB^Bur^, suggesting that these sequence changes are not required for resistance breaking. This is consistent with the hypothesis, put forward by Amrao *et al*. [23], that the avirulence determinant for the CLCuD resistance in cotton introduced during the late 1990s was the C2 protein—based upon the finding that the virus involved in resistance breaking (CLCuBuV) lacks an intact C2 gene. Nevertheless, there are distinct differences between the βC1 sequences of CLCuMB occurring in the Punjab and Sindh for the majority of isolates. The reason for this is unclear, but could be due to the founder effect, with sequence changes selected for due to a requirement other than resistance breaking, spreading throughout a region. This in turn would suggest that the selection pressures in the Punjab and Sindh differed, the difference possibly being the distinct cotton varieties in the two regions. In light of the possibility of the betasatellite not being directly involved in resistance breaking in cotton, it would seem expedient to, in future, use CLCuBuV (specifically the truncated C2 gene; [23]) as the genetic marker of the resistance breaking virus complex rather than CLCuMB^Bur^ (specifically the ToLCB recombination).

The function of the SCR of betasatellites remains a mystery. The position of the SCR in betasatellites is analogous to the position of the common region (CR) of bipartite begomoviruses [7]. The CR is a sequence shared between the two components of the genomes of bipartite begomoviruses which sits in the intergenic region, encompasses the promoter driving complementary-sense gene expression, sequence motifs (known as iterons; [44]) that are high affinity binding sites for the virus encoded replication-associated protein (Rep; a rolling-circle replication-initiator protein; [15]) as well as a predicted hairpin structure (containing the nonanucleotide sequence TAATATTAC, as part of the loop, that is nicked by Rep to initiate rolling-circle replication; [15]). The SCR also sits in an intergenic region but does not contain promoter elements, these instead being situated in the sequence upstream of the A-rich sequence ahead of the complementary sense βC1 gene [45], or iterons. The iterons or rather, iteron-like sequences, instead appear to be situated in the sequence between the SCR and the A-rich region [39,41]. The SCR does contain a predicted hairpin structure with a nonanucleotide sequence which presumably is required for the helper virus-encoded Rep to initiate satellite DNA replication, although this has not yet been proven experimentally. The position of the SCR, surrounding the origin of replication, may suggest a role in interaction with host factors involved in DNA replication. In light of this, it is difficult to know what benefits, if any, there are from recombination between betasatellites within the SCR, such as those for CLCuMB^Bur^ and CLCuMB^Sha^ other than to suggest that this may improve betasatellite trans-replication.

Although it is possible that each of these distinct CLCuMB types/strains has arisen independently by recombination (inserting ToLCB sequence and possibly also sequences of unknown origin into the SCR and also between SCR and A-rich region), a far more plausible explanation is that after an initial (large) insertion, the sequence of the recombinant fragment was sequentially reduced by recombination with CLCuMB^Mul^. Since resistance breaking, CLCuMB^Mul^ has not been encountered in cultivated cotton in the Punjab, appears not to be present in Sindh and has recently become rare in cultivated cotton in India (an example being CLCuMB-[IN:Bih:10] HM461864). Nevertheless, this strain of CLCuMB remains in the environment in weeds, non-cultivated cotton species and probably other crops such as tomato [46,47]. These sequence changes are thus more likely to be occurring in a host species other than cotton.

The finding that many of the features of the CLCuMB isolates from Sindh are shared with CLCuMB isolates from India (the full-length fragment upstream of the ToLCB sequences and the significant changes in the sequences between the SCR and the A-rich region), but not with isolates from the Punjab, suggests that there is more movement of CLCuMB, and thus presumably also the virus(es), between north western India and Sindh than previously thought. Why CLCuMB in the Punjab should be distinct is unclear. Possibly, the widespread adoption of resistant cotton varieties in the Punjab, which was not the case in either Sindh or India, has selected for distinct variants/strains of the satellite. In light of the evidence suggesting that the satellite is not involved (directly) with resistance breaking, the selection mechanism on CLCuMB in the Punjab may be indirect, mediated by the virus. This is supported by the finding that the range of CLCuD-associated viruses in the three regions differ: no diversity in the Punjab (only CLCuBuV present in cotton [23]); some diversity in India (CLCuBuV and CLCuRaV identified [4]); and some diversity in Sindh (*Cotton leaf curl Gezira virus*, *Cotton leaf curl Kokhran virus* and *Cotton leaf curl Shahdadpur virus* [25,27]). If this is the case, then it is likely that the diversity of the satellite is limited by the virus, which in turn is limited by the cotton variety.

The study presented shows that the CLCuD betasatellite in south Asia is evolving quite rapidly, although the forces driving this are unclear. A better understanding of virus-satellite interactions is required before a full understanding of the effects will be forthcoming. In addition the results have highlighted a high gene flow between the three regions—Punjab, Sindh and northwestern India. A better understanding of the movement of the pathogen might be useful in future efforts to control the disease; particularly should there be further epidemics.

## Supplementary Files

Supplementary File 1 Supplementary Information (PDF, 361 KB)
