# Supplementary material for: Regional Changes in the Sequence of Cotton Leaf Curl Multan Betasatellite
Source: Viruses. 2014 May 23;6(5):2186–203. doi: 10.3390/v6052186 (PMC4036549; doi:10.3390/v6052186)
Supplement: Supplementary File 1 — Supplementary Information (PDF, 361 KB) [file viruses-06-02186-s001.pdf]

Supplementary Information

Figure S1. RDP analysis of Cotton leaf curl Multan betasatellite sequences. Shown are linear representations of the sequences of selected CLCuMB isolates that are representative of the sub-groups discussed in the text. The origins of sequences (either from Cotton leaf curl Multan betasatellite strain Burewala ($CLCuMB^{Mu]l}$ or Tomato leaf curl betasatellite (ToLCB)) are shown as coloured boxes relative to the approximate positions of betasatellite features satellite conserved region (SCR), βC1 gene (βC1) and adenine-rich region (A-rich) shown at the top of the figure.

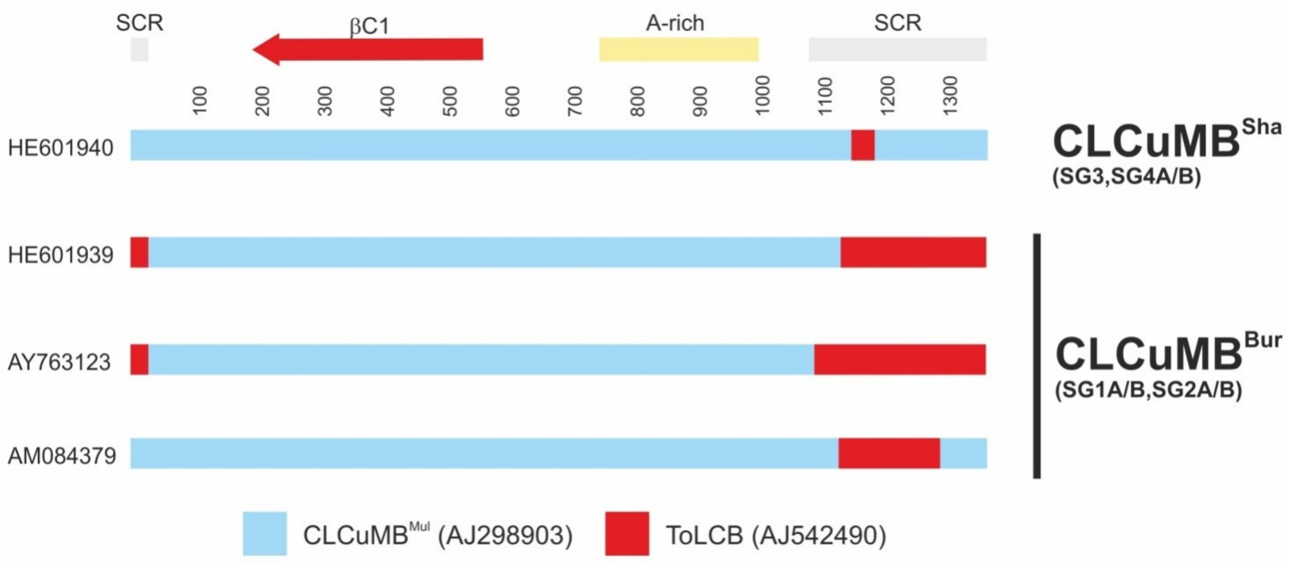

Table S1. Recombination analysis of selected Cotton leaf curl Multan betasatellite isolates using the RDP 3.0 program.

Event no.	Breakpoint positions		Recombinant sequence *	Minor parent *	Major parent *	Detection methods (*p* value)					
	Begin	End				RDP	GENECONV	BootScan	MaxChi	Chimera	Siscan
1	1155	1185	CLCuMB^{Sha} [HE601940]	ToLCB [AJ542490]	CLCuMB^{Mul} [AJ298903]	-	3.140×10^{-05}	-	-	-	-
2	1128	26	CLCuMB^{Bur} [HE601939]	ToLCB [AJ542490]	CLCuMB^{Mul} [AJ298903]	8.288×10^{-31}	2.504×10^{-34}	4.171×10^{-32}	2.368×10^{-11}	5.777×10^{-12}	-
3	1087	20	CLCuMB^{Bur} [AY763123]	ToLCB [AJ542490]	CLCuMB^{Mul} AJ298903	6.083×10^{-18}	2.574×10^{-13}	2.069×10^{-15}	2.313×10^{-04}	1.845×10^{-06}	-
4	1142	1292	CLCuMB^{Bur} [AM084379]	ToLCB [AJ542490]	CLCuMB^{Mul} [AJ298903]	2.917×10^{-29}	2.273×10^{-22}	4.080×10^{-26}	5.392×10^{-10}	3.753×10^{-11}	-

* The betasatellite species/strains are given as Tomato leaf curl betasatellite (ToLCB), Cotton leaf curl Multan betasatellite strains Multan (CLCuMB^{Mul}), Burewala (CLCuMB^{Bur}) and Shahdadpur (CLCuMB^{Sha}). In each case the relevant database accession number is given.
